# Supplementary material for: VapC12 ribonuclease toxin modulates host immune response during Mycobacterium tuberculosis infection
Source: Front Immunol. 2024 Mar 7;15:1302163. doi: 10.3389/fimmu.2024.1302163 (PMC10955575; doi:10.3389/fimmu.2024.1302163)
Supplement: Supplementary file 3 [file Presentation_1.pdf]

Figure S1:

A.

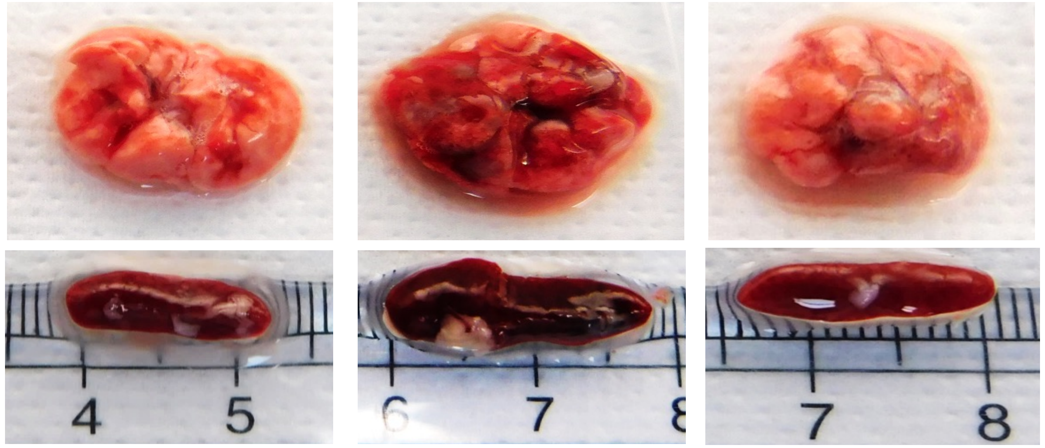

H37Rv **Δ*vapC12*** **Δ*vapC12:vapBC12***

Figure S2

A.

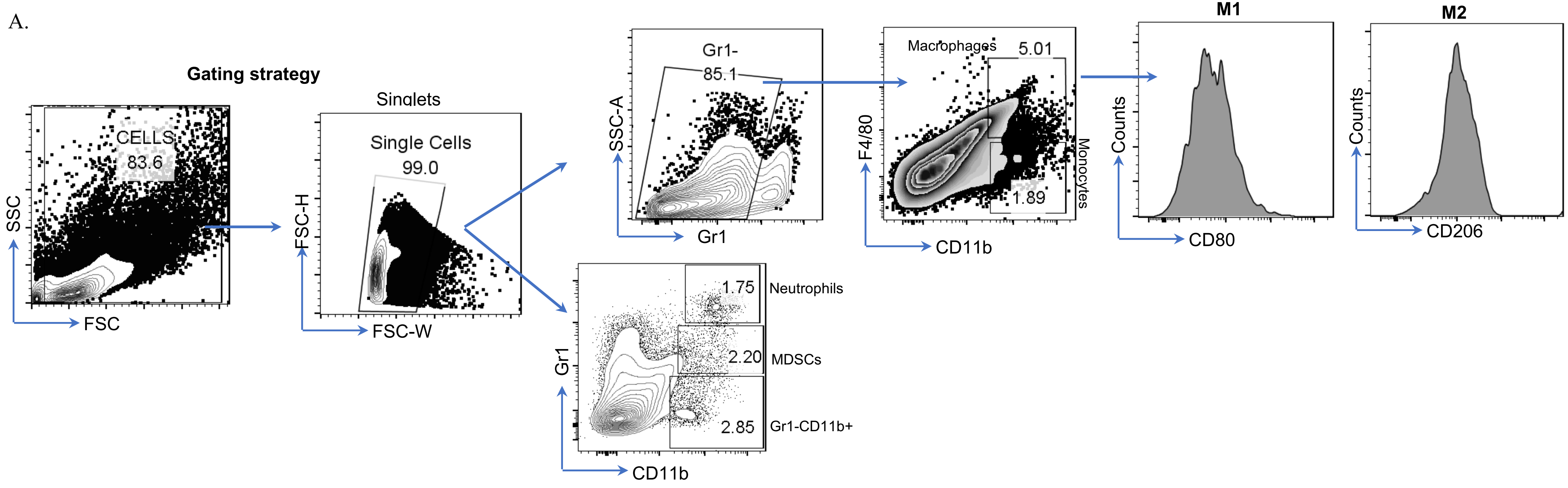
Gating strategy
CELLS
83.6
SSC
FSC
Singlets
Single Cells
99.0
FSC-H
FSC-W
Gr1-
85.1
SSC-A
Gr1
1.75
Neutrophils
2.20
MDSCs
2.85
Gr1-CD11b+
Gr1
CD11b
Macrophages
5.01
1.89
Monocytes
F4/80
CD11b
M1
Counts
CD80
M2
Counts
CD206

Figure S3

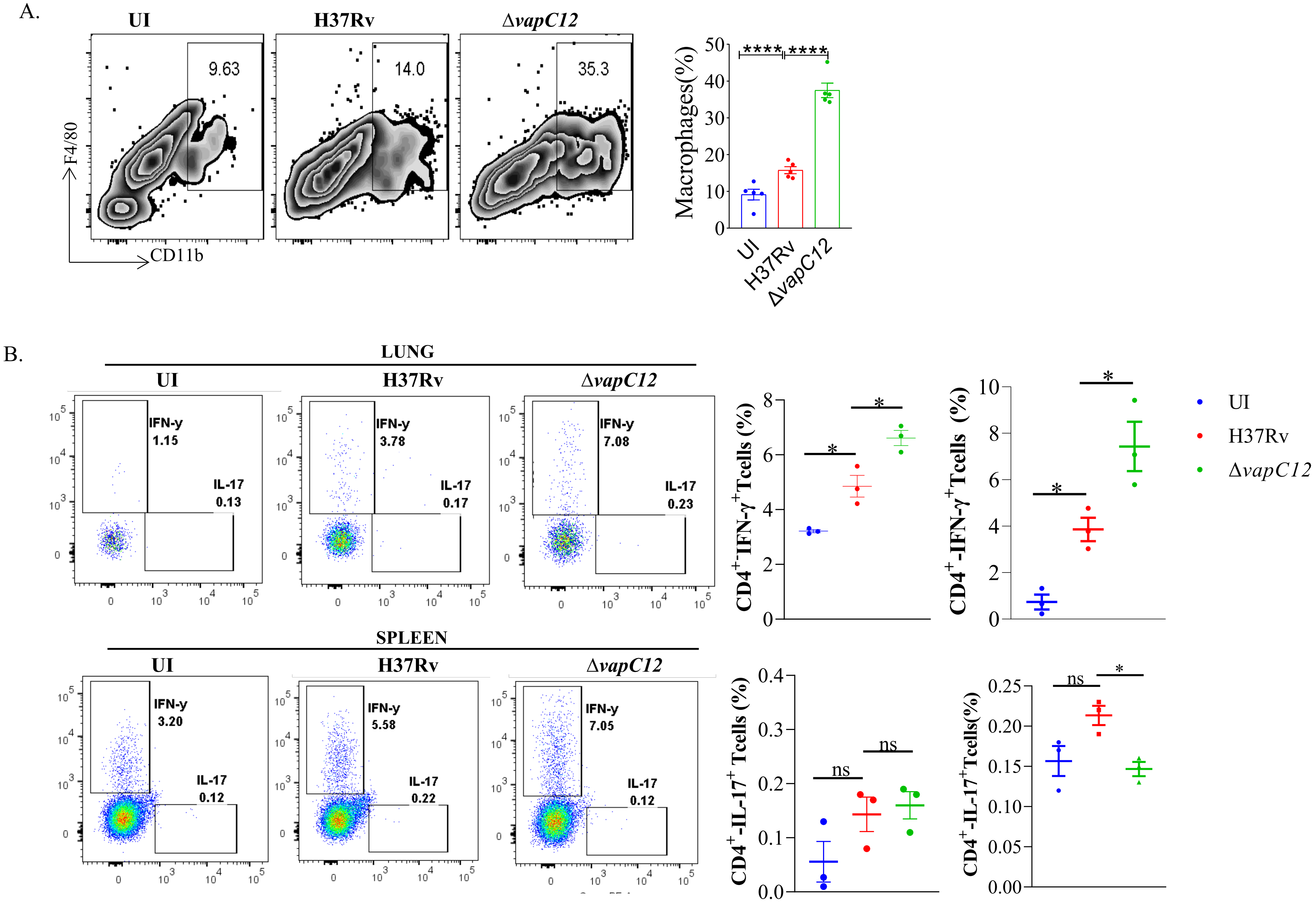
A.
UI
H37Rv
ΔvapC12
9.63
14.0
35.3
F4/80
CD11b
Macrophages(%)
****
****
UI
H37Rv
ΔvapC12
B.
LUNG
UI
H37Rv
ΔvapC12
IFN-γ
1.15
3.78
7.08
IL-17
0.13
0.17
0.23
CD4+IFN-γ+Tcells (%)
CD4+-IFN-γ+Tcells (%)
*
UI
H37Rv
ΔvapC12
SPLEEN
UI
H37Rv
ΔvapC12
IFN-γ
3.20
5.58
7.05
IL-17
0.12
0.22
0.12
CD4+-IL-17+ Tcells (%)
CD4+-IL-17+Tcells(%)
ns

Figure S4

A.

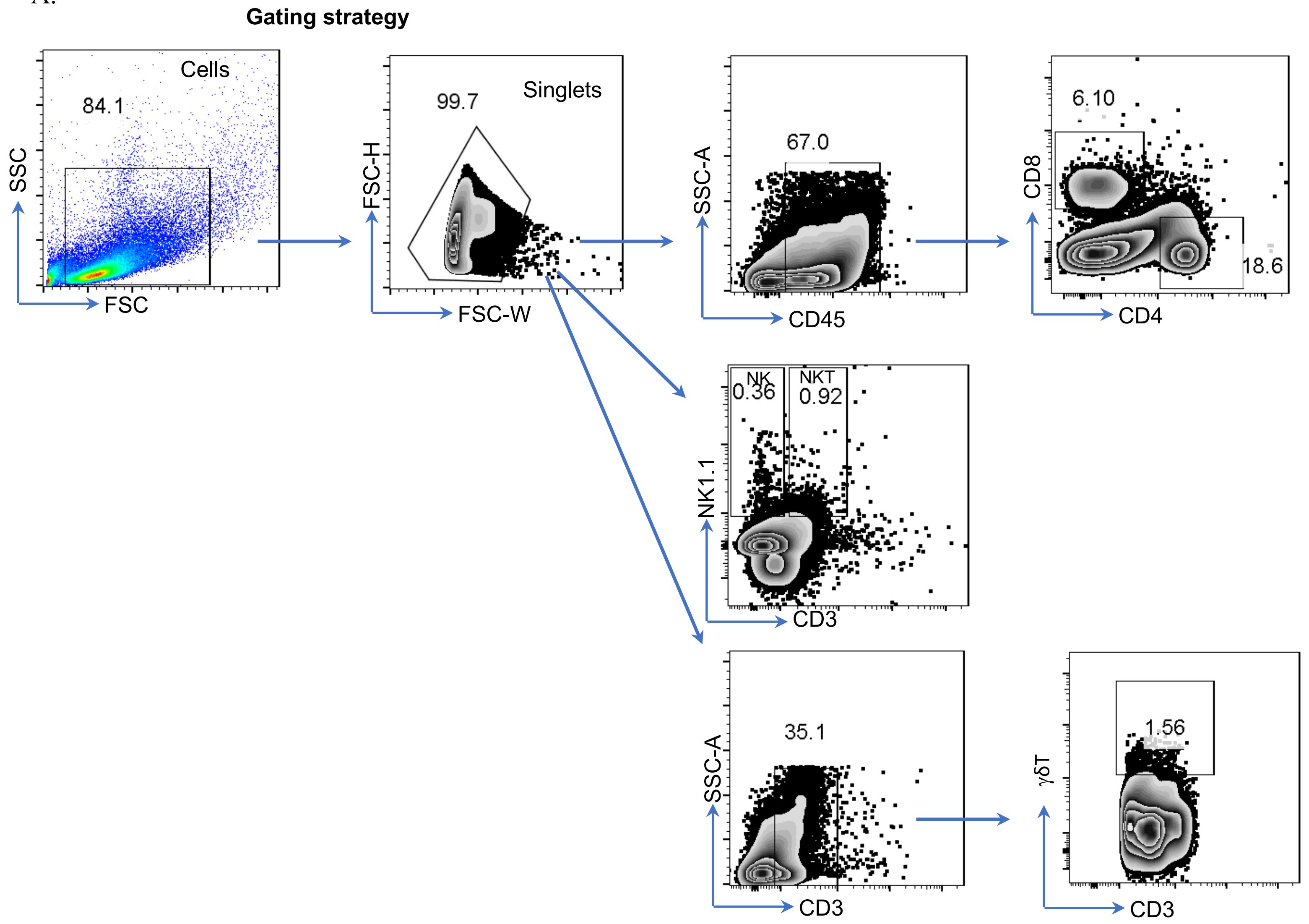
Gating strategy
Cells
84.1
SSC
FSC
99.7
Singlets
FSC-H
FSC-W
67.0
SSC-A
CD45
6.10
18.6
CD8
CD4
NK
0.36
NKT
0.92
NK1.1
CD3
35.1
SSC-A
CD3
1.56
γδT
CD3

Figure S5

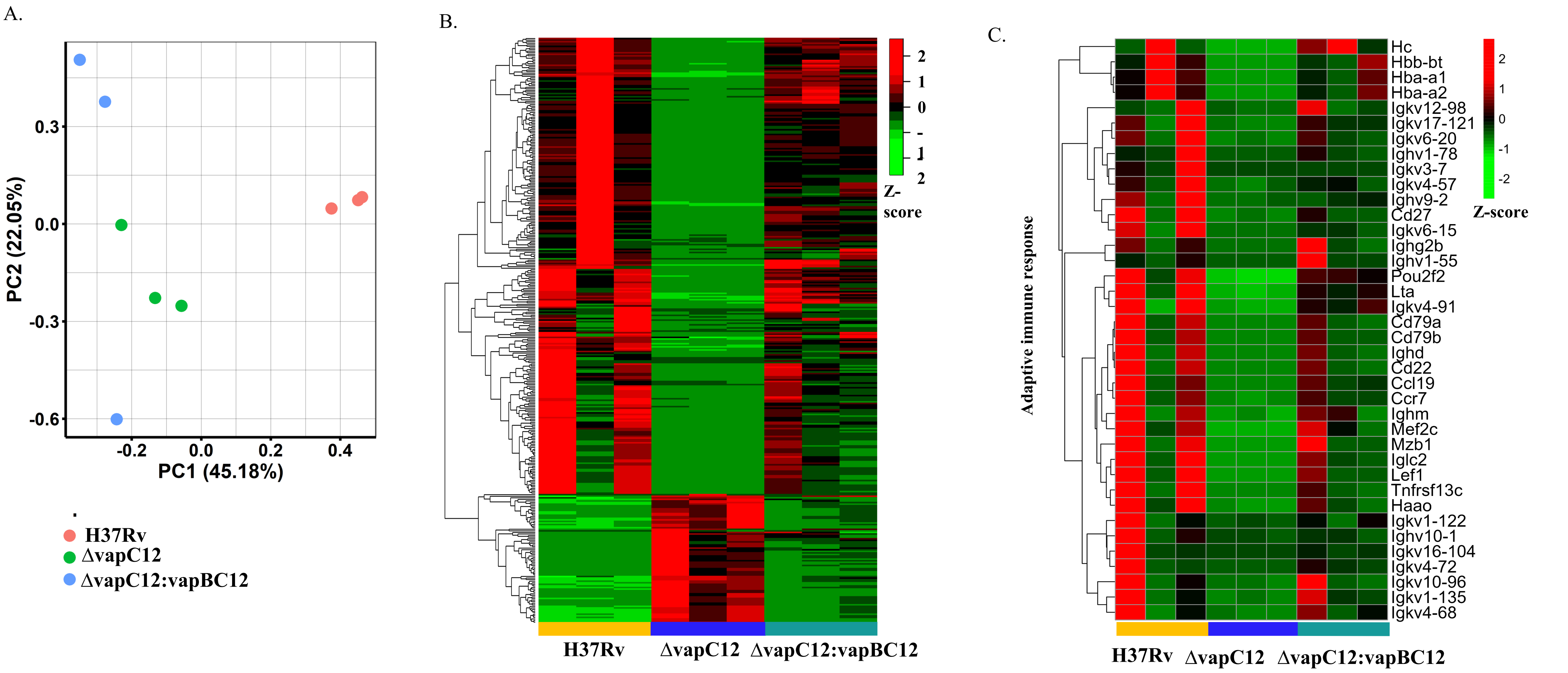
A.
PC2 (22.05%)
PC1 (45.18%)
0.3
0.0
-0.3
-0.6
-0.2
0.0
0.2
0.4
H37Rv
ΔvapC12
ΔvapC12:vapBC12
B.
Z-score
2
1
0
H37Rv
ΔvapC12
ΔvapC12:vapBC12
C.
Adaptive immune response
Hc
Hbb-bt
Hba-a1
Hba-a2
Igkv12-98
Igkv17-121
Igkv6-20
Ighv1-78
Igkv3-7
Igkv4-57
Ighv9-2
Cd27
Igkv6-15
Ighg2b
Ighv1-55
Pou2f2
Lta
Igkv4-91
Cd79a
Cd79b
Ighd
Cd22
Ccl19
Ccr7
Ighm
Mef2c
Mzb1
Iglc2
Lef1
Tnfrsf13c
Haao
Igkv1-122
Ighv10-1
Igkv16-104
Igkv4-72
Igkv10-96
Igkv1-135
Igkv4-68
2
1
0
-1
-2
Z-score
H37Rv
ΔvapC12
ΔvapC12:vapBC12

Figure S6

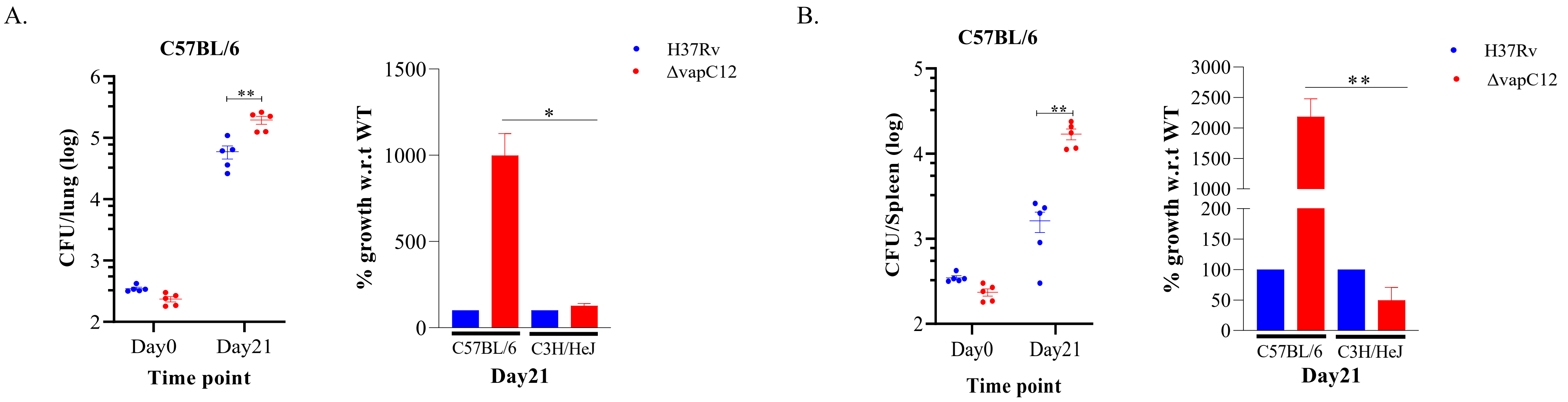
A.
C57BL/6
CFU/lung (log)
6
5
4
3
2
**
Day0
Day21
Time point
% growth w.r.t WT
1500
1000
500
0
*
C57BL/6
C3H/HeJ
Day21
H37Rv
ΔvapC12
B.
C57BL/6
CFU/Spleen (log)
5
4
3
2
**
Day0
Day21
Time point
% growth w.r.t WT
3000
2500
2000
1500
1000
200
150
100
50
0
**
C57BL/6
C3H/HeJ
Day21
H37Rv
ΔvapC12

Supplementary Figures

Figure S1: (A) Gross pathology of the lungs and spleen of mice infected with various strains of *M. tuberculosis* at 8 weeks post infection**.**

Figure S2: (A) Gating strategy for Macrophages (M1 and M2 population), MDSCs, neutrophils and monocytes population.

Figure S3: (A) Zebra plot and its representative bar graph showing the percentage frequency of macrophages (Gr1-CD11b+F4/80+) harvested from lungs. Data are shown as mean ± SEM (n=5 animals per group). Data were analyzed using the one-way ANOVA followed by Tukey's multiple comparison tests (*P <0.05, **P < 0.01, ***P<0.0005, ****P<0.0001).

(B) FACS plot and Bar graphs indicating the percentage frequency of $CD4^{+}$-IFN-γ T cells and $CD4^{+}$-IL-17^{+} T cells in the lung and spleen. Data were analyzed using FlowJo and shown as mean ± SEM (n=3 animals per group). Data were analyzed using the one-way ANOVA test followed by Tukey's multiple comparison test (*P <0.05, **P < 0.01, ***p<0.0005, ****p<0.0001).

Figure S4: (A) Gating strategy for innate (NK, NKT and γδT) and adaptive immune cell population ($CD4^{+}$ and $CD8^{+}$ CELLS).

Figure S5: (A) Principal component analysis (PCA) of the transcriptomic data obtained from lung tissues of mice infected with WT, Δ*vapC12* and complement strain after 4 weeks of infection. The data shown are obtained from three biological replicates.

(B) Heatmap representing the expression patterns of the 372 differentially expressed genes (DEG) obtained from comparing gene expression profiles from mice infected with parental and ΔvapC12 strain of *M. tuberculosis*. Data were Z-score transformed. The data shown are obtained from three biological replicates, and the names of the corresponding strains are mentioned at the bottom of the figure. DEGs were obtained using the fold change cut-off value of 2 and FDR-adjusted p-value < 0.05.

(C) Heatmap representing the expression levels of the 38 down-regulated genes involved in the adaptive immune response. Data were Z-score transformed and the name of the genes are displayed on the right. The names of the corresponding infected strains for each sample are mentioned at the bottom of the figure.

Figure S6: (A and B) C57BL/6 mice were aerosol infected with H37Rv and Δ *vapC12.* Scatter plot showing bacterial load in the (A) lungs and (B) spleen of mice infected with H37Rv and *ΔvapC12* at indicated time points. Each group constituted five mice per time point. Bar graph depicts percentage plot showing growth of *vapC12* mutant with respect to WT in C57BL/6 and C3H/HeJ. Data plotted represent the mean ± SEM. Significant differences observed between groups are indicated. Data were analyzed using the Mann–Whitney test with **P < 0.01 and *P <0.05.
